# Comparison of IGF-1 Serum and Nutritional Status in Pediatric Ventricular Septal Defect: A Case–Control Study

**DOI:** 10.3390/children13060785

**Published:** 2026-06-04

**Authors:** Taufiq Hidayat, Irwanto Irwanto, Ali Rohman, Shabrina Nur Imanina, Ayurveda Zaynabila Heriqbaldi, Bagas Triambodo, Afrizal Alif Azzam Muhyiddin, Achmad Ari Pratama, Mahrus A. Rahman, I Ketut Alit Utamayasa, Nur Syamsiatul Fajar, Mochamad Amin

**Affiliations:** 1 Department of Child Health, Faculty of Medicine, Universitas Airlangga, Surabaya 60132, Indonesia; irwanto@fk.unair.ac.id (I.I.); bagas.triambodo-2025@fk.unair.ac.id (B.T.); afrizal.alif.azzam-2024@fk.unair.ac.id (A.A.A.M.); achmad.ari.pratama-2024@fk.unair.ac.id (A.A.P.); mahrus.a@fk.unair.ac.id (M.A.R.); ketut.alit.utamayasa@fk.unair.ac.id (I.K.A.U.); 2 Division of Cardiology, Department of Child Health, Dr. Soetomo General Hospital, Surabaya 60286, Indonesia; 3 Universitas Airlangga Hospital, Surabaya 60115, Indonesia; 4 Department of Chemistry, Faculty of Science and Technology, Universitas Airlangga, Surabaya 60115, Indonesia; alirohman@fst.unair.ac.id; 5 Faculty of Medicine, Universitas Airlangga, Surabaya 60132, Indonesia; shabrinanuriman@gmail.com (S.N.I.); hana.zaynabila@gmail.com (A.Z.H.); 6 Tropical Disease Diagnostic Center, Institute of Tropical Disease, Universitas Airlangga, Surabaya 60286, Indonesia; fajar@staf.unair.ac.id (N.S.F.); moch.amin@staf.unair.ac.id (M.A.)

**Keywords:** ventricular septal defect, IGF-1, nutritional status, congenital heart disease

## Abstract

**Highlights:**

**What are the main findings?**

- Children with ventricular septal defect demonstrated significantly lower serum IGF-1 concentrations compared with healthy controls.
- Moderate and severe wasting were more prevalent among children with ventricular septal defect
- Lower IGF-1 levels were associated with poorer nutritional status.

**What are the implications of the main findings?**

- IGF-1 may reflect nutritional and metabolic impairment in pediatric ventricular septal defect.
- Combined nutritional assessment and biomarker evaluation may support earlier identification of children at risk for growth failure.

**Abstract:**

**Background/Objectives**: Ventricular septal defect (VSD) is the most common congenital heart disease in children and is often associated with growth impairment and malnutrition. Increased metabolic demand, feeding difficulties, and recurrent infections contribute to poor nutritional status. Insulin-like growth factor-1 (IGF-1), a key mediator of growth hormone activity, reflects nutritional and metabolic conditions. Previous studies have evaluated endocrine and growth abnormalities in heterogeneous congenital heart disease populations. However, data specifically examining the relationship between serum IGF-1 levels and nutritional status in isolated pediatric ventricular septal defect remain limited, particularly in Southeast Asian populations. **Methods**: The single centre observational case–control study was conducted at Dr. Soetomo Hospital, Surabaya, involving 110 children (55 VSD patients and 55 healthy controls). VSD diagnosis was confirmed by echocardiography. Nutritional status was assessed using WHO anthropometric criteria. Serum IGF-1 levels were measured using ELISA. Statistical analyses compared IGF-1 levels between groups and across nutritional categories. **Results**: Moderate and severe wasting were more common in the VSD group. Median IGF-1 levels were significantly lower in VSD patients compared to controls (5.18 vs. 21.4 ng/mL; *p* < 0.001). A positive association between IGF-1 levels and nutritional status was observed. **Conclusions**: Children with VSD have poorer nutritional status and significantly lower IGF-1 levels compared to healthy controls. This association may be explained by the dysregulation of the growth hormone–IGF-1 axis. IGF-1 may complement nutritional assessment for identifying and monitoring growth impairment and guiding early nutritional interventions in pediatric VSD.

## 1. Introduction

Ventricular septal defect (VSD) is the most common congenital heart defect in children and remains an important contributor to growth impairment and morbidity in pediatric populations. Malnutrition and growth failure are prevalent in children with VSD, with high rates of underweight, stunting, and wasting reported across congenital heart disease (CHD) populations [1,2]. Nutritional compromise arises from the hemodynamic burden of chronic left-to-right shunting, which increases metabolic demand, impairs feeding efficiency, and heightens susceptibility to recurrent respiratory infections, all of which accelerate nutritional deterioration and failure to thrive [1,2,3]. Prior studies have demonstrated that hemodynamic severity, including shunt magnitude and pulmonary-to-systemic flow ratio, is a primary determinant of growth failure in children with VSD [4]. Although surgical correction improves weight gain, recovery of height and full somatic normalization may remain suboptimal, particularly among infants with severe preoperative malnutrition [5]. Poor nutritional status in children with CHD has been consistently associated with prolonged hospitalization, increased perioperative risk, and adverse developmental outcomes, underscoring the importance of early and systematic nutritional assessment [3].

Nutritional assessment in children with VSD requires a graded rather than binary classification. The WHO growth standards distinguish severe wasting, moderate wasting, normal, overweight, and obesity, each carrying distinct clinical and hormonal implications. Severe wasting, corresponding anthropometrically to severe acute malnutrition (SAM), is associated with maximal suppression of IGF-1 alongside paradoxically elevated GH, reflecting profound GH resistance in a catabolic state [6,7]. Moderate wasting, by contrast, represents partial and potentially reversible GH–IGF-1 axis suppression, while children with normal nutritional status serve as an essential within-disease reference. This graded approach is particularly relevant in VSD, where IGF-1 suppression may reflect both the hemodynamic burden and the cumulative depth of nutritional deprivation [8,9]. The present study therefore applied WHO 2006 and 2007 Child Growth Standard with full nutritional subcategorization to enable a more precise characterization of IGF-1 dysregulation across the nutritional spectrum in children with isolated VSD [10,11].

Prior investigations have explored IGF-1 and growth impairment in children with congenital heart disease. A previous study demonstrated reduced IGF-1 levels and impaired growth parameters in children with isolated VSD, suggesting dysregulation of the GH–IGF-1 axis in acyanotic CHD [8]. Subsequent studies also reported altered IGF-1 levels in broader CHD populations, although findings regarding its relationship with nutritional status have remained inconsistent [12,13]. However, previous studies evaluated heterogeneous CHD populations combining cyanotic and acyanotic lesions or utilized older anthropometric references [13,14]. Limiting the ability to determine the specific relationship between IGF-1 and WHO-standardized nutritional status in isolated VSD patients.

Evidence focusing specifically on serum IGF-1 and WHO-standardized nutritional assessment in pediatric isolated VSD populations therefore remains limited, particularly in Southeast Asian settings that may have different nutritional patterns, healthcare access, timing of congenital heart disease diagnosis, and prevalence of childhood undernutrition from high-income settings where most endocrine growth studies have been conducted. Therefore, this study aimed to compare serum IGF-1 levels and nutritional status between children with isolated VSD and healthy controls, and to evaluate the correlation between serum IGF-1 and WHO-standardized anthropometric parameters in pediatric VSD patients.

## 2. Materials and Methods

This study is a case-control study in children with VSD in the pediatric cardiology and inpatient clinic of Child Health Department of Dr. Soetomo Hospital, Surabaya. The protocol was approved by the institutional ethics committee (N.0980/KEPK/V/2024). The sample size was calculated using a case-control formula (α = 0.05, β = 0.20, power = 80%). The required sample size was estimated to be 49–55 subjects per group. Therefore, 55 cases and 55 controls were included.

Inclusion criteria cases were children with isolated VSD confirmed by pediatric cardiologist assessment and transthoracic echocardiography. VSD characteristics, including defect type, defect size, and the presence of pulmonary hypertension, were assessed and classified by a pediatric cardiologist based on 2D transthoracic echocardiographic findings. Cardiomegaly was evaluated based on chest radiograph interpretation. Controls were children without structural heart disease on clinical evaluation. Exclusion criteria for both groups were: major syndromic diagnoses known to affect cardiac structure, prior cardiac surgery (palliative or corrective), or incomplete core variables required for analysis.

Demographic and anthropometric variables were abstracted from medical records. Anthropometric measurements were obtained using standardized procedures. Body weight was measured using a calibrated digital scale, and height/length was measured using a stadiometer or length board. Nutritional status was categorized as obese, overweight, normal, moderate wasting, severe wasting, based on WHO 2006 and 2007 classification thresholds. Peripheral blood samples were collected. Serum IGF-1 levels were measured using a commercially available enzyme-linked immunosorbent assay (ELISA) kit (Elabscience™ Human IGF-1 ELISA Kit, Catalog No. E-EL-H0086; Elabscience Biotechnology Inc., Wuhan, China).

Statistical analyses were performed using IBM SPSS Statistics Version 22. Normality of continuous variables was assessed using the Shapiro-Wilk test. Normally distributed variables were expressed as mean ± standard deviation (SD) and compared between groups using the independent samples *t*-test (Welch correction). Non-normally distributed variables were expressed as median (interquartile range, IQR) and compared using the Mann-Whitney U test. Categorical variables were expressed as frequency and percentage (*n*, %) and compared using Fisher’s exact test. The correlation between serum IGF-1 levels and nutritional status indices was assessed using Spearman rank correlation. Multiple linear regression analysis was performed to identify independent predictors of serum IGF-1 levels, with age, nutritional status, VSD type, pulmonary hypertension, and cardiomegaly included as covariates. A *p*-value of <0.05 was considered statistically significant.

## 3. Results

A total of 110 children were included in this study, comprising 55 patients with ventricular septal defect and 55 healthy controls.

### 3.1. Characteristics of the Study Subject

Table 1 summarizes the characteristics of study subjects across both groups. The mean age of children in the case group was 2.07 ± 1.28 years, which was comparable to that of the control group (1.85 ± 0.83 years), with no statistically significant difference (*p* = 0.986). Gender distribution was also similar between groups. These findings indicate that the two groups were well-matched in terms of age and gender, minimizing potential confounding from these variables.

Anthropometric assessment demonstrated poorer nutritional parameters in the case group compared to controls. Children in the case group had a significantly lower median weight-for-age z-score (WAZ) (−1.94 [IQR: −2.73 to −1.28]) compared to controls (−0.20 [IQR: −1.28 to 0.67]) (*p* < 0.001), indicating a greater tendency toward underweight status. The mean height-for-age z-score (HAZ) was also significantly lower in the case group (−1.46 ± 1.87) than in controls (0.27 ± 1.95) (*p* < 0.001), suggesting a higher prevalence of impaired linear growth or stunting among children with VSD.

The weight-for-height z-score (WHZ) was substantially lower in the case group (−2.33 [IQR: −2.52 to −1.07]) than in controls (−0.52 [IQR: −2.39 to 0.07]) (*p* = 0.002), reflecting a greater degree of wasting. This comparison was conducted on available data only, as WHZ is not applicable in children older than five years (case: *n* = 46; control: *n* = 54). The median BMI-for-age z-score (BAZ) was also markedly lower in the case group (−2.27 [IQR: −2.54 to −0.87]) compared with controls (−0.57 [IQR: −2.25 to 0.16]) (*p* = 0.004), further indicating poorer overall nutritional status among cases.

Regarding nutritional categories, moderate and severe wasting were more frequently observed in the case group, whereas normal nutritional status predominated in controls; however, this difference did not reach statistical significance (*p* = 0.074).

Table 2 presents the clinical characteristics of VSD in the case group (*n* = 55). Regarding VSD type, the majority of children had perimembranosus VSD (37, 67.3%), followed by SADC (10, 18.2%) and muscular type (8, 14.5%). In terms of defect size, most cases presented with moderate-sized VSD (36, 65.5%), while small and large defects accounted for 20% (*n* = 11) and 14.5% (*n* = 8) of cases, respectively.

Pulmonary hypertension (PH) was present in 19 children (34.5%), with mild PH being the most common severity (12, 21.8%), followed by moderate (4, 7.3%) and severe (3, 5.5%). The remaining 36 children (65.5%) had no evidence of pulmonary hypertension. Cardiomegaly was identified in 15 cases (27.3%), while the majority did not exhibit cardiac enlargement (40, 72.7%).

### 3.2. Comparison of Serum IGF-1 Levels

Serum IGF-1 levels were significantly lower in children with VSD compared to health controls (*p* < 0.001) (Table 3).

### 3.3. IGF Levels According to Nutritional Status

Children classified as malnourished demonstrated significantly lower IGF-1 levels than those with normal nutritional status (Table 4).

Spearman correlation analysis demonstrated a significant positive correlation between IGF levels and nutritional status in both groups (Table 5). In the control group, a moderate positive correlation was observed (ρ = 0.510, *p* < 0.001), whereas the case group showed a stronger positive correlation (ρ = 0.635, *p* < 0.001).

### 3.4. Multiple Linear Regression Analysis of Factors Associated with IGF Levels in Cases Group

Multiple linear regression analysis was performed to evaluate the associations between age, nutritional status, defect classification, pulmonary hypertension, and cardiomegaly with IGF levels (Table 6). Based on the ANOVA results, the regression model was statistically significant (F(5,49) = 9.742, *p* < 0.001), indicating that the independent variables collectively contributed to the prediction of IGF levels. The model explained 49.9% of the variance in IGF levels (R^2^ = 0.499; adjusted R^2^ = 0.447). Among the variables included in the model, only nutritional status showed a significant independent association with IGF levels (B = 5.383, β = 0.625, *p* < 0.001; 95% CI: 3.384–7.382). Age, defect classification, pulmonary hypertension, and cardiomegaly were not significantly associated with IGF levels (all *p* > 0.05).

## 4. Discussion

This study demonstrated that children with ventricular septal defect exhibited significantly poorer nutritional status and lower serum IGF-1 levels compared with healthy controls. Growth impairment remains a major clinical concern in children with congenital heart disease, particularly among those with hemodynamically significant shunt lesions. Children with VSD frequently experience increased metabolic demand secondary to chronic volume overload, recurrent respiratory infections, feeding fatigue, and inefficient caloric utilization, all of which may contribute to inadequate growth and undernutrition [15,16]. Studies in Iran reported by Tabib et al. (2019) and metanalysis by Diao et al. (2021) also reported an increased prevalence of underweight, stunting, and failure to thrive in congenital heart disease populations, emphasizing that nutritional compromise represents an important determinant of both clinical outcomes and perioperative recovery [5,17,18].

The positive correlation between IGF-1 levels and nutritional status observed in this study suggests that IGF-1 reflects both nutritional and metabolic status is also consistent with study from Barton et al. (1993) and Dinleyici (2007) that serum IGF-I was significantly lower in infants with CHD than in controls [12,19]. IGF-1 synthesis is influenced by protein intake, hepatic function, and overall energy balance, making it a sensitive indicator of growth potential and nutritional adequacy [8]. Children with inadequate nutrition exhibit reduced IGF-1 levels, which improve following nutritional rehabilitation [20]. Insulin-like growth factor-1 (IGF-1) is a key mediator of growth hormone (GH) activity and plays a central role in somatic growth, cellular metabolism, and cardiovascular development [21]. In the context of chronic cardiac disease, the sustained hemodynamic burden of left-to-right volume overload may induce a state of GH resistance, in which adequate GH secretion fails to produce sufficient hepatic IGF-1 synthesis, a mechanism distinct from that observed in cyanotic lesions, where hypoxia-driven pathways predominate [8,22,23]. This GH resistance leads to alterations in IGF-binding proteins, particularly IGFBP-3, the principal carrier of IGF-1 in circulation, further disrupting the GH–IGF-1 axis and impairing linear growth [21,23]. Beyond its role in somatic growth, IGF-1 directly supports myocardial growth and adaptive remodeling in response to hemodynamic stress, establishing a bidirectional relationship between growth axis function and cardiac physiology [21]. Chronic inflammatory activation has been shown to disrupt the growth hormone (GH)–IGF-1 axis through increased pro-inflammatory cytokines, particularly interleukin (IL)-6, IL-1β, and tumor necrosis factor-α (TNF-α), which can induce GH resistance by decreasing GH receptor signaling and suppressing hepatic IGF-1 synthesis [24]. Decreased serum IGF-1 further impairs chondrocyte proliferation and growth plate activity, contributing to linear growth failure. In chronic pediatric diseases, persistent metabolic stress coupled with impaired anabolic signaling can further exacerbate catabolism and nutritional depletion, creating a reciprocal cycle of inflammation, malnutrition, and growth impairment [24,25]. This mechanism supports the findings of this study, where anthropometric status in the VSD group different significantly from that in the healthy group.

Beyond nutritional status, hemodynamic burden may also contribute to variability in IGF-1 concentrations. Defect classification demonstrated a borderline association with IGF-1 concentrations, suggesting that structural severity may partly influence endocrine and metabolic adaptation, whereas pulmonary hypertension was not independently associated with IGF-1 levels. Soliman et al. (2002) previously reported associations between echocardiographic severity and endocrine-growth parameters in children with VSD, larger or hemodynamically significant VSDs are induced cardiac workload and hypermetabolic state characterized by increased resting metabolic rate and oxygen consumption. Despite apparently normal food intake, the combination of elevated metabolic demands and increased cardiac work reduces the energy available for growth, thereby contributing to undernutrition and growth impairment [8]. While more recent findings suggest that impaired growth and lower IGF-1 concentrations in congenital heart disease may improve following correction of cardiovascular abnormalities [14]. However, because only a limited number of patients exhibited moderate-to-severe pulmonary hypertension in the present study, these findings should be interpreted cautiously.

Several limitations should be considered when interpreting findings in this study. First, the cross-sectional design precludes causal inference regarding the directionality of the relationship between nutritional status and IGF-1 concentrations. Although the case and control groups were age-matched, IGF-1 concentrations were not normalized to age- and sex-specific reference ranges, which may have influenced the comparability of values across the developmental spectrum represented in this cohort. Important determinants of IGF-1 physiology such as growth hormone concentration, inflammatory biomarkers, liver function status, feeding intolerance severity, and IGFBP-3 concentrations, were not measured. Therefore, mechanistic interpretations regarding endocrine dysregulation should be interpreted cautiously. Although defect size classification and pulmonary hypertension status were included, more detailed hemodynamic parameters such as shunt fraction, pulmonary artery pressure quantification, and heart failure severity were not comprehensively analyzed and may partly explain residual variability in IGF-1 concentrations. Finally, the single-center design and relatively limited sample size may restrict generalizability.

Despite these limitations, this study provides additional evidence supporting the close relationship between nutritional status and circulating IGF-1 concentrations in children with isolated VSD. Nutritional status remained the strongest independent determinant of IGF-1 levels, suggesting that IGF-1 biomarkers may complement routine nutritional assessment in identifying children at risk for growth impairment. Future longitudinal studies combining hemodynamic characterization, inflammatory biomarkers, and endocrine profiles are recommended in the future.

## 5. Conclusions

Children with ventricular septal defect demonstrated significantly poorer nutritional status and lower serum IGF-1 concentrations compared with healthy controls. The positive association between IGF-1 levels and nutritional status suggests a close relationship between nutritional adequacy and endocrine growth regulation in pediatric VSD. Although the underlying mechanisms were not directly evaluated, reduced IGF-1 concentrations may reflect the combined influence of nutritional compromise and chronic cardio-metabolic stress associated with congenital heart disease.

These findings support the potential role of IGF-1 as a complementary biomarker reflecting nutritional and growth-related status in children with VSD. Routine nutritional assessment, complemented by IGF-1 evaluation, may assist in identifying children at greater risk for growth impairment. Further longitudinal studies are warranted to determine the prognostic value of IGF-1 and to clarify the biological mechanisms underlying growth dysregulation in pediatric VSD.

## Figures and Tables

**Table 1:** Characteristics of Case and Control Subject.

Characteristic	Case (*n* = 55)	Control (*n* = 55)	*p*-Value
Age (years), Mean ± SD	2.07 ± 1.28	1.85 ± 0.83	0.986
Gender, *n* (%)			
Male	27 (49.09)	28 (50.91)	1.000
Female	28 (50.91)	27 (49.09)	
Weight (kg), Median (IQR)	9.5 (7.4–13)	16 (14–18)	<0.001
Height (cm), Median (IQR)	80 (71.5–100)	105 (100–112.25)	<0.001
Anthropometric Status			
WAZ, Median (IQR)	−1.94 (−2.73–−1.28)	−0.2 (−1.28–0.67)	<0.001
HAZ, Mean ± SD	−1.46 ± 1.87	0.27 ± 1.95	<0.001
WHZ *, Median (IQR)	−2.33 (−2.52–−1.07)	−0.52 (−2.39–0.07)	0.002
BAZ, Median (IQR)	−2.27 (−2.54–−0.87)	−0.57 (−2.25–0.16)	0.004
Nutritional Status, n (%)			
Severe wasting	1 (1.82)	0 (0.00)	0.074
Moderate wasting	26 (47.27)	17 (30.91)	
Normal	27 (49.09)	37 (67.27)	
Overweight	1 (1.82)	0 (0.00)	
Obese	0 (0.00)	1 (1.82)	

* WHZ not applicable for children > 5 years (case: *n* = 46; control: *n* = 54). *p* < 0.05 considered statistically significant.

**Table 2:** Characteristics of VSD in the Case Group.

VSD Type	*N* (%)
Perimembranosus	37 (67.3)
Muscular	8 (14.5)
SADC	10 (18.2)
**VSD Size**	
Small	11 (20)
Moderate	36 (65.5)
Large	8 (14.5)
**Pulmonary Hypertension**	
Mild	12 (21.8)
Moderate	4 (7.3)
Severe	2 (3.6)
None	37 (67.3)
**Cardiomegaly**	
Yes	15 (27.3)
None	40 (72.7)

**Table 3:** Comparison of Serum IGF-1 Levels in Case and Control.

Parameter	Cases (*n* = 55)	Controls (*n* = 55)	U	*p*-Value
IGF 1, ng/mL, Median (IQR)	5.18 (3.18–12.10)	21.4 (10.17–24.78)	519.0	<0.001

**Table 4:** IGF-1 Levels According to Nutritional Status in Case and Control.

Nutritional Status	Cases			Control		
	*N* (%)	IGF-1 (ng/mL)	*p*-Value	*N* (%)	IGF-1 (ng/mL)	*p*-Value
Severe wasting	1 (1.82)	2.72	<0.001	0 (0.00)	-	<0.001
Moderate wasting	26 (47.27)	3.41 (1.90–4.55)		17 (30.91)	5.24 (4.06–8.93)	
Normal	27 (49.09)	12.01 (8.65–13.14)		37 (67.27)	23.45 (17.80–25.24)	
Overweight	1 (1.82)	8.31		0 (0.00)	-	
Obese	0 (0.00)	-		1 (1.82)	13.9	

IGF-1 levels presented as Median (IQR); single values reported for groups with *n* = 1. *p*-values derived from Spearman rank correlation. *p* < 0.05 considered statistically significant.

**Table 5:** Correlation between IGF and Nutritional Status using Spearman Correlation Analysis.

Group	Variables	*n*	Spearman’s Rho (ρ)	*p*-Value
Control	IGF vs. Nutritional Status	55	0.510	<0.001
Case	IGF vs. Nutritional Status	55	0.635	<0.001

**Table 6:** Multiple Linear Regression Analysis.

Variable	B	Standardized β	*p*-Value	95% CI
Age	−0.348	−0.164	0.144	−0.818–0.122
Nutritional Status	5.383	0.625	<0.0001	3.384–7.382
Defect Classification	−1.788	−0.214	0.051	−3.587–0.010
Pulmonary Hypertension	0.313	0.081	0.530	−0.683–1.310
Cardiomegaly	0.409	0.037	0.748	−2.136–2.954

Model statistics: R = 0.706; R^2^ = 0.499; Adjusted R^2^ = 0.447; F(5,49) = 9.742; *p* < 0.001.

## Data Availability

The data presented in this study are available on request from the corresponding author. The data are not publicly available due to ethical and privacy restrictions related to patient confidentiality.

## Abbreviations

The following abbreviations are used in this manuscript:

VSD	Ventricular Septal Defect
CHD	Congenital Heart Disease
WAZ	Weight-for-age z-score
HAZ	Height-for-age z-score
WHZ	Weight-for-height z-score
BAZ	Body mass index for age z-score
IGF-1	Insulin-Like Growth Factor-1
GH	Growth Hormone
IGFBP-3	Insulin-Like Growth Factor-Binding Protein 3
IL-6	Interleukin (IL)-6
IL-1β	Interleukin-1 beta
TNF-α	Tumor Necrosis Factor-α
SAM	Severe acute malnutrition
SADC	Subarterial doubly committed (VSD type)
WHO	World Health Organization
ELISA	Enzyme-Linked Immunosorbent Assay
SD	Standard Deviation
IQR	Interquartile Range
